# Resilience and mobile phone addiction among adolescent athletes: a chain-mediated model of anxiety and depression

**DOI:** 10.3389/fpsyt.2025.1646150

**Published:** 2025-11-28

**Authors:** Feng Tian, Rong Zhang, Fengbo Liu

**Affiliations:** 1 College of Physical and Education, Zhengzhou University of Light Industry, Zhengzhou, China; 2 School of Education, Cangzhou Normal University, Cangzhou, China; 3 School of Psychology,Beijing Sport University, Beijing, China

**Keywords:** resilience, MPA, adolescent athletes, emotional mechanisms, anxiety, depression

## Abstract

**Background:**

Mobile phone addiction (MPA) has become a growing concern among adolescents, particularly within the group of adolescent athletes in high-pressure competitive environments. resilience may serve as a protective factor, but the underlying emotional mechanisms remain unclear. This study aimed to examine the mediating roles of anxiety and depression in the relationship between resilience and MPA among adolescent athletes.

**Methods:**

A total of 401 participants (aged 13–19) were recruited from a sports secondary school in Henan Province, China. Resilience, anxiety, depression, and MPA were measured using validated self-report scales. Structural equation modeling and bootstrap analysis (5,000 resamples) were conducted using Mplus 8.3.

**Results:**

Resilience was not significantly associated with MPA in the direct path. For the indirect path, the pathway from resilience to MPA through depression was significant, whereas the pathway through anxiety was not. And a significant chain mediation was found from resilience to MPA through both anxiety and depression. The total indirect effect was significant, while the direct effect was not.

**Conclusion:**

Resilience is associated with lower levels of MPA in adolescents, primarily through its impact on depressive symptoms, with anxiety playing an indirect role through depression. These findings suggest that interventions promoting resilience and reducing depression may help mitigate problematic phone use in youth.

## 1 Introduction

As time progresses and technology develops, mobile phones are gradually becoming an integral part of life (1). This increasing reliance often leads to a series of problems when individuals overuse their mobile phones, which can interfere with normal functioning (2). This issue is particularly pronounced among underage groups, who are more susceptible to MPA, which can significantly impact their daily lives (3). In scientific field, MPA, also referred to as problematic mobile phone use, is increasingly recognized as a behavioral addiction marked by excessive, uncontrolled, and compulsive use of mobile devices that interferes with various aspects of daily life (4). It is characterized by symptoms such as preoccupation with phone-related activities, a persistent inability to reduce usage despite awareness of negative consequences, and significant distress or functional impairment when the device is inaccessible (5). The research by Pew Research Center shows that, relative to other age groups, over 95% of American adolescents own a personal phone, and 40% of them feel them spend much time on it (6). As smartphone ownership becomes nearly universal among adolescents and young adults, the boundary between functional use and addictive behavior grows increasingly blurred. The reason is that unlike other behavioral addictions such as internet gaming or gambling, phone use is unique in its omnipresence and integration into daily routines of adolescents used for socializing, academic tasks, entertainment, and emotional escape (7). The consequences of MPA can be serious and may lead to various negative impact. Some of empirical studies have linked it to reduced academic engagement, decreased productivity, impaired interpersonal relationships, and sleep disturbances, particularly due to bedtime screen exposure and nocturnal awakenings triggered by phone notifications (8). From a neuropsychological perspective, it shares core features with other forms of addiction, such as impulsivity, diminished executive control, and heightened sensitivity in some conditions (9). These behavioral and neurocognitive markers highlight the necessity of addressing mobile phone overuse, especially among adolescent group. These not merely as a habit but as a potential public health concern requiring targeted intervention strategies. In order to address this addictive behavior, it is important to identify which emotional factors are more strongly associated with it. Research has shown that athletes are typically spend more time on training, and their levels of MPA are significantly lower compared to non-athlete who rarely engage in physical activity (10). However, MPA is exactly existing with athletes, such as Instagram addiction (11), media loyalty (12) and PUBG addiction (13). Previous research has clearly indicated that there are two major aspects associated with MPA among athletes: one is negative (e.g., anxiety, distraction, disengagement), and the other is positive (e.g., self-regulation, social connectedness) (14). Exploring the complex correlations between MPA and psychological mechanisms is important for developing an influential MPA model for adolescent athlete group.

### 1.1 The relationship between resilience and MPA

According to the Emotion Regulation Deficit Theory, MPA is clearly associated with deficits in emotion regulation, as individuals who lack this ability are more likely to develop addictive behaviors (15). Individuals with insufficient emotion regulation skills are more likely to engage in maladaptive coping strategies, such as excessive smartphone use, to relieve negative emotions (16). These temporary strategies can develop into behavioral addictions over time.

Resilience, as a protective trait, enhances one’s ability to manage stress and regulate emotions effectively, thereby reducing the likelihood of engaging in emotion-driven addictive behaviors like MPA (17). And resilience has been identified as a key component of mental health and is associated with positive psychological outcomes, particularly during periods of adversity (18). Resilience encompasses both emotional and cognitive components, and its level serves as an effective indicator for assessing an individual’s psychological adaptability when facing changing environments or challenging situations (19). Findings from resilience research suggest that the presence of protective factors may buffer the effects of stress, thereby promoting physical health and preventing the development of psychopathology, even in the face of significant adversity (20). But on the other hand, although resilience originates from the field of developmental psychopathology, there is still no universally established theoretical framework for it (21). From the perspective of physical activity, both meta-analyses and empirical studies have shown that physical activity can serve as a modifiable factor influencing resilience (22). This is partly because physical activity enhances top-down control over bottom-up processes in the brain, thereby promoting self-regulation. By strengthening specific brain regions and large-scale neural circuits, it improves emotional and behavioral regulation, serving as a protective buffer against mental health problems and fostering resilience during this vulnerable developmental period (23). It can be concluded that resilience plays a pivotal role in supporting individuals who are regularly active, thereby reducing MPA, especially among athletes. However, we aim to explore the complex correlations between resilience and MPA on this group, as ample evidence shows that a portion of this population still experiences high levels of MPA (24). Among the many potential factors, an individual’s psychological adaptability in the face of adversity emerges as a crucial indicator worth observing. Moreover, previous research appears to have insufficiently explored the role of resilience in influencing MPA among adolescent athletes (25). Our aim is to extend prior findings. Although existing research suggests that physical activity may help alleviate anxiety, it is clear that it is not the only effective intervention (26). Identifying specific intervention factors can help clarify one aspect of the complex web of related influences. The resilience and MPA status of adolescent athletes remain insufficiently studied. This group typically engages in relatively high levels of physical activity, and based on existing theories, their resilience may also be comparatively stronger. We proposed the following research question:

H1: Resilience is negatively associated with MPA.

### 1.2 The mediating role of anxiety

Anxiety is regarded as an emotional state in response to the anticipation of potential negative events in the future (27). It is also one of the most common mental disorders (28). Its main symptoms include verbal-subjective manifestations such as worry, overt motor acts such as avoidance, and somato-visceral activity such as muscle tension (29). Common types of anxiety disorders include social anxiety disorder (13% lifetime prevalence), generalized anxiety disorder (6.2% lifetime prevalence), panic disorder (5.2% lifetime prevalence), and agoraphobia (2.6% lifetime prevalence), which frequently co-occurs with panic disorder (30). Potential comorbidities include cardiovascular diseases, gastrointestinal diseases, pulmonary diseases, cancer, chronic pain, and migraine headaches (31). Anxiety disorders are chronic conditions, and research has found that many patients experience symptoms for several years before receiving any therapeutic intervention (32). They typically onset during childhood or adolescence and persist into adulthood (33). The median age of onset is as early as 11 years old. The onset of generalized anxiety disorder is most commonly reported to occur during childhood and adolescence (34). Notably, students at this age are typically in the stage of compulsory education, where anxiety is often frequent, widespread, and intense within the educational environment (35). Moreover, anxiety experienced during this period can lead to a range of problems in life after graduation (36). Researchers have extensively examined the relationship between anxiety disorders and various contextual evaluations. The cognitive consequences of test anxiety can lead to a decline in working memory capacity, thereby impairing the completion of learning tasks (37). Social anxiety can negatively affect individuals in educational, social, and interpersonal domains (38). For children, anxiety disorders may result in chronic school refusal, which can subsequently lead to significant social and academic impairments (39). For adolescents, anxiety disorders can lead to a decline in academic performance and have a detrimental impact on social functioning (28). In adults, anxiety disorders exhibit relatively high comorbidity rates with other psychiatric disorders, including depression, substance abuse/dependence, and attention-deficit hyperactivity disorder (ADHD) (40). Therefore, early intervention and management of anxiety can help alleviate its associated dysfunction and distress.

A review of the literature reveals that the underlying mechanisms influencing adolescent anxiety can be categorized into three main types. The first involves biological factors. In addition to the previously discussed dysregulation of the hypothalamic-pituitary-adrenal (HPA) axis, another critical factor is the autonomic nervous system (ANS), which consists of the sympathetic and parasympathetic branches (41). Together, these systems regulate cardiovascular responses: the sympathetic nervous system stimulates the body’s reaction to stress, while the parasympathetic nervous system inhibits it (42). Low vagal (parasympathetic) responsiveness and a low threshold for sympathetic activation are considered potential mechanisms in the development of anxiety and depression (43). The second category involves individual and environmental factors. Studies have found that, within certain subgroups, girls are more likely than boys to develop anxiety and depression disorders (44). Environmental factors include low family socioeconomic status (SES) and parental internalizing problems, both of which have been associated with increased risk of anxiety (45). Parents with anxiety or depression disorders often have limited social resources, which diminishes their ability to help their children cope with stressful social situations, thereby increasing the risk of anxiety and depression in their offspring. In addition, early adverse experiences, such as bereavement, parental divorce, physical abuse, and stressful life events, also contribute to the development of anxiety disorders (46). The third category involves potential risk factors at the intersection of various domains and psychology. For example, the biological consequences of obesity may affect the HPA axis and lead to negative body image perceptions and dissatisfaction due to one’s physical appearance. This in turn, can result in low self-esteem and social anxiety, thereby increasing the likelihood of adolescents developing anxiety (47). This is particularly important among adolescents, as many experience the onset of anxiety symptoms for the first time during high school (48). Early onset is often associated with the development of mental disorders in adulthood (49). From a functional perspective, anxiety often triggers avoidance behaviors and attentional biases toward threat (50). Anxious individuals may turn to mobile devices as a form of safety-seeking or distraction from worry (51). Based on it, we proposed the following research questions:

H2: Anxiety mediates the relationship between resilience and MPA.

### 1.3 The mediating role of depression

Depression is typically conceptualized as a persistent emotional state characterized by feelings of sadness, hopelessness, and a loss of interest or pleasure in previously enjoyed activities (52). It encompasses not only affective symptoms, but also cognitive impairments such as negative self-evaluation, indecisiveness, and recurrent rumination. Additionally, somatic symptoms, such as fatigue, changes in sleep and appetite, and psychomotor retardation, further compound its impact on functioning (53). Depression is thus a multidimensional construct that exerts broad impairments across emotional, cognitive, and physical domains. Although anxiety and depression are highly comorbid and share overlapping features such as negative affect, their core phenomenological and neurobiological profiles differ substantially (54). Anxiety is predominantly future-oriented, characterized by excessive worry, hypervigilance, and physiological arousal in anticipation of potential threats (55). In contrast, depression is more focused on past or present experiences, marked by low affect, anhedonia, and a pervasive sense of helplessness or resignation. Neurocognitively, anxiety tends to involve hyperactivation of the amygdala and related fear circuits, while depression is more associated with hypoactivation in reward-related brain areas such as the ventral striatum and prefrontal cortex (56). Previous studies have already confirmed a significant association between resilience and depression; however, whether this relationship remains robust within the adolescent athlete population warrants further investigation.

Research has shown that anxiety and depression can significantly affect adolescents’ mental health and overall well-being (57). Given these theoretical, clinical, and neurobiological distinctions, it is conceptually justified to treat anxiety and depression as separate yet potentially interacting mediators in psychological models. This approach allows for a more nuanced examination of how distinct forms of emotional distress may differentially mediate the association between resilience and maladaptive behaviors (58). Depression results in withdrawal, cognitive slowing, and motivational deficits. These distinctions have important implications for behavioral outcomes such as MPA. For example, whereas depressed individuals may use them for passive escape or mood regulation in response to low energy or motivation (59).

A growing body of research underscores the complex interplay between psychological distress and MPA (60). Numerous studies have shown that individuals experiencing heightened levels of anxiety, depression, or emotional dysregulation are significantly more susceptible to problematic phone use (61). For these individuals, mobile phones often serve as an accessible and immediate coping mechanism, offering temporary relief from negative affect through distraction, reassurance-seeking, or virtual social connection. However, such reliance may lead to maladaptive coping patterns, reinforcing avoidance behaviors and ultimately exacerbating psychological symptoms. Among adolescents, a population particularly vulnerable due to ongoing emotional development and identity formation of the relationship appears especially pronounced. Frequent phone use may temporarily alleviate feelings of loneliness or academic stress, but over time, excessive dependence on virtual interactions can erode self-esteem, heighten social anxiety, and impair real-world social functioning (62). Moreover, longitudinal studies suggest a bidirectional relationship: while poor mental health predicts higher levels of MPA, the addiction itself can also lead to a decline in psychological well-being, forming a reinforcing cycle of dysfunction (63). These findings underscore the need for an integrated mental health framework when investigating the antecedents and consequences of MPA, particularly in youth-focused prevention and intervention efforts.

Does resilience play a key role in the relationship between depression and MPA? To what extent does resilience influence the relationship between depression and MPA? We proposed the following research questions:

H3: Depression mediates the relationship between resilience and MPA.

### 1.4 The chain mediating effect of anxiety and depression

Recent research has increasingly highlighted the psychological challenges faced by adolescents in the digital age, with MPA emerging as a prevalent behavioral concern (62). Resilience has been widely recognized as a protective factor against emotional distress, such as anxiety and depression, both of which are positively linked to the development of MPA (64). While numerous studies have explored these variables in general adolescent populations, little is known about their interactions within the unique context of adolescent athletes. Adolescent athletes are often assumed to benefit from structured training routines and enhanced physical fitness, which may contribute to better emotional regulation (65). However, this group is also subject to unique stressors, such as performance pressure, intense schedules, and limited time autonomy (66). These factors may make them equally—if not more—vulnerable to psychological distress and maladaptive coping behaviors like MPA (24). Given these dual characteristics, it remains unclear whether the protective role of resilience functions similarly among adolescent athletes, and whether anxiety and depression mediate this relationship in the same manner as in the general adolescent population. Therefore, the present study aims to examine the chain relationships among resilience, anxiety, depression, and MPA in adolescent athletes. Specifically, we test a chain mediation model in which anxiety and depression sequentially mediate the relationship between resilience and MPA. And the hypothesis is:

H4: Anxiety and depression sequentially mediate the relationship between resilience and MPA.

**Figure 1:**
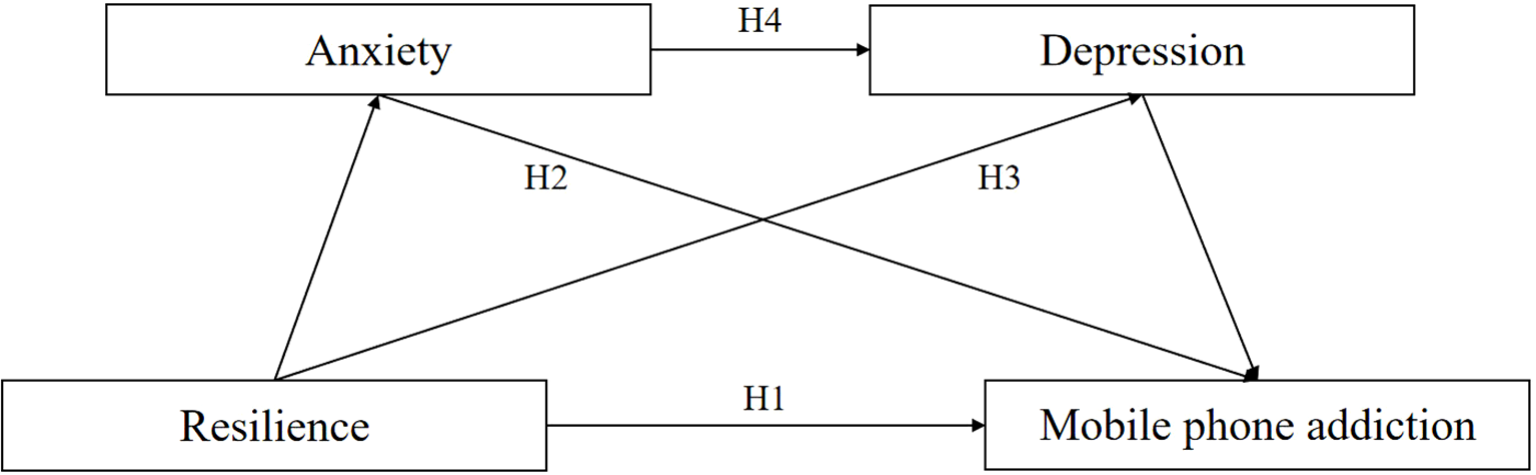
Hypothesized model.

Based on the above, we propose the following model (**Figure 1**):

## 2 Methods

### 2.1 Participants

The study was reviewed and approved by the Research Ethics Committee of Zhengzhou University of Light Industry. An online screening questionnaire was used to assess eligibility. Those who met the inclusion criteria were invited to participate in the study. Prior to data collection, informed consent was obtained from all participants, and confidentiality and anonymity were fully ensured. For minors, parental consent was also obtained before the start of the investigation. All procedures were conducted in accordance with ethical guidelines.

A convenience sampling method was used in this study. A total of 500 questionnaires were distributed, and 410 were returned. After excluding 9 invalid responses due to incomplete or inconsistent answers, 401 valid questionnaires were retained for analysis, yielding an effective response rate of 80.2%. The participants were adolescents aged 13 to 19 years (M = 14.56, SD = 0.86), recruited from a sports-focused secondary school in Henan Province, China. The sample consisted predominantly of male students (approximately 77%), and the majority were Han Chinese (over 95%). All participants received regular training in various athletic disciplines as part of their school curriculum. Most participants were under the age of 18.

### 2.2 Instruments

#### 2.2.1 Connor-Davidson resilience scale

Resilience was assessed using the 10-item Chinese version of the Connor-Davidson Resilience Scale (CD-RISC-10) (67), revised by Chinese researchers for use among adolescent athletes. The scale comprises 10 items rated on a 5-point Likert scale (1 = “not true at all” to 5 = “true nearly all the time”). Higher scores reflect higher levels of psychological resilience. In the current study, the scale demonstrated excellent internal consistency (Cronbach’s α = 0.932).

#### 2.2.2 Generalized anxiety disorder scale

Anxiety symptoms were measured using the 7-item Generalized Anxiety Disorder Scale (GAD-7) modified version, originally developed by Spitzer et al. based on the DSM-IV criteria (68). The scale has been translated into Chinese and psychometrically validated by local researchers for use among Chinese populations (69), including adolescent groups. It is a unidimensional scale rated on a 3-point Likert scale, with higher total scores indicating greater levels of anxiety. A sample item is “Feeling afraid as if something awful might happen.” In the present study, the scale demonstrated good internal consistency (Cronbach’s α = 0.866).

#### 2.2.3 Center for epidemiologic studies depression scale

Depressive symptoms were assessed using the Chinese version of the Center for Epidemiologic Studies Depression Scale (CES-D), adapted by Chinese researchers (70). The scale includes 20 items measuring the frequency of depressive symptoms experienced during the past week. Items are rated on a 4-point Likert scale, with higher total scores indicating more severe depressive symptoms. The scale has demonstrated good reliability and validity in Chinese adolescent populations. In the present study, the internal consistency was acceptable (Cronbach’s α = 0.893).

#### 2.2.4 Mobile phone addiction tendency scale

MPA tendency was measured using the Mobile Phone Addiction Tendency Scale (MPATS), developed by Xiong Jie et al. (71). The scale consists of 16 items rated on a 5-point Likert scale (1 = “strongly disagree” to 5 = “strongly agree”), with higher scores indicating a greater tendency toward MPA. It comprises four dimensions: withdrawal symptoms (e.g., “I feel uneasy when I can’t use my phone”), salience behavior, social soothing, and mood change. The scale has been widely used in Chinese adolescent and young adult populations, and has shown good psychometric properties. In the present study, the internal consistency of the scale was acceptable (Cronbach’s α = 0.931).

### 2.3 Statistical analysis

As a initial step, we examined the descriptive statistics and bivariate associations among the core variables by computing Pearson correlation coefficients in SPSS 26.0. For mediation analysis, the bootstrap method was adopted based on Hayes’ recommendation. Unlike the Sobel test, the bootstrap method estimates the sampling distribution directly without assuming normality, offering greater statistical power and a lower Type I error rate (72). Therefore, this study employed the bootstrap method with 5,000 resamples to estimate the 95% confidence intervals of the mediation effects. All mediation analyses were conducted using Mplus version 8.3.

According to all indicators were ordinal Likert-type items, we additionally estimated the model using WLSMV with all items treated as categorical, as a sensitivity analysis to verify the stability of the model.

## 3 Results

### 3.1 Common method bias check

Since all variables in this study were derived from self-reported questionnaires, common method bias (CMB) may pose a significant threat to discriminant validity. To mitigate potential CMB, several procedural remedies were implemented, including anonymous responses, counterbalanced item sequencing, and standardized administration protocols. We assessed common method bias using Harman’s single-factor test, performing an unrotated exploratory factor analysis on all questionnaire items (73). The analysis revealed 9 components with eigenvalues greater than 1, collectively explaining 61.69% of the total variance. Notably, the first unrotated principal component accounted for 28.81% of the variance, which is below the critical threshold of 50%, indicating that no single factor dominated the variance structure. This result suggests that common method bias is unlikely to pose a serious threat to the validity of the findings.

### 3.2 Discrimination validity check

We conducted confirmatory factor analysis (CFA) to examine whether the measurement model of the adopted scales holds in our adolescent sample.

We used maximum likelihood estimation with robust standard errors (MLR) to handle non-normality. All items were retained without parceling to avoid information loss. Model modifications were not applied to maintain theoretical purity. The model demonstrated the overall model acceptable fit: χ² (1319) = 2760.36, *p* <.001, RMSEA = 0.052, CFI = 0.843, TLI = 0.836, SRMR = 0.065. Although the CFI and TLI values were slightly below the conventional threshold of.90, this is not uncommon for models with complex structures, and the other fit indices fell within acceptable ranges. We further test all items factor loadings (**Supplementary Table S1**), while most standardized of them exceeded the recommended threshold of 0.40, three items fell below this cutoff. Given that the present study employed a widely validated instrument, these items were retained for theoretical consistency, though their weaker performance is noted as a limitation. In conclusion, this model supporting the distinctiveness of the constructs.

### 3.3 Descriptive statistics and correlation analysis

**Table 1** presents the means, standard deviations, and intercorrelations among the key study variables: gender, age, ethnicity, resilience, anxiety, depression, and MPA. Resilience was negatively correlated with anxiety (r = -0.43, p < 0.001), depression (r = -0.53, *p* < 0.001), and MPA (r = -0.33, p < 0.001), indicating that higher resilience is associated with better psychological adjustment and lower behavioral risk. Anxiety and depression were positively correlated (r = 0.62, *p* < 0.001), and both showed significant positive associations with MPA (r = 0.37 and r = 0.47, respectively, both *p* < 0.001). These findings support the hypothesized associations and provide justification for subsequent mediation analysis. Although age was modestly correlated with several variables, these effects were small and are not considered confounding.

**Table 1:** Descriptive statistics and correlation analysis of variable results.

Variable	Mean (S.D.)	1.	2.	3.	4.	5.	6.	7.
1. Gender	1.23 (0.42)	–						
2. Age	14.56 (0.86)	-0.082	–					
3. Nation	1.03 (0.17)	0.077	0.022	–				
4. Resilience	3.55 (0.76)	0.029	0.143**	0.080	–			
5. Anxiety	1.77 (0.43)	-0.014	-0.113*	-0.087	-0.428***	–		
6. Depression	1.68 (0.45)	-0.059	-0.126*	-0.058	-0.533***	0.615***	–	
7. MPA	2.77 (0.77)	-0.022	-0.103*	0.010	-0.327***	0.372***	0.473***	–

*p < 0.05, **p < 0.01, ***p < 0.001.

Gender, age, grade nation were categorical variables codes as: gender (1 = male, 2 = female), nation(1 = Han, 2 = ethnic minority).

### 3.4 Mediator effect analysis

We tested the path hypotheses (H1, H2, H3, H4) using structural equation modeling (SEM) with maximum likelihood (ML) estimation and 5000 bootstrap resamples. This approach allows simultaneous estimation of multiple pathways and accounts for measurement error in latent constructs. Standardized path coefficients and bias-corrected 95% confidence intervals were reported.

**Table 2** presents the indirect and total effects estimated via structural equation modeling with 5000 bootstrap resamples. The direct path from resilience to MPA was non-significant (β = -0.135, 95% CI [-0.281, -0.012]), not supporting H1. The indirect path from resilience to MPA via depression (H3) was statistically significant (β = -0.076, 95% CI [-0.132, -0.021]), as was the chained mediation via anxiety and depression (H4; β = -0.095, 95% CI [-0.141, -0.049]). However, the simple mediation pathway via anxiety alone (H3) was not statistically significant (β = -0.051, 95% CI [-0.141, 0.040]). Overall, the total indirect effect was significant (β = -0.222, 95% CI [-0.294, -0.149]), the direct effect was non-significant (β = -0.135, 95% CI [-0.281, 0.012]), indicating that the association between resilience and MPA is largely explained through depression and the chained pathway. These findings support H3 and H4 but not H1 and H2.

**Table 2:** Results of chain mediating effect of Resilience on MPA.

Effect	Effect size (β)	Boot SE	95% CI [LL, UL]	Proportion mediated
Total effect	-0.356	0.059	[-0.472, -0.240]	—
Direct effect	-0.135	0.075	[-0.281, 0.012]	—
Total indirect effect	-0.222	0.037	[-0.294, -0.149]	62.42%
Resilience → Anxiety → MPA	-0.051	0.046	[-0.141, 0.040]	14.33%
Resilience → Depression → MPA	-0.076	0.028	[-0.132, -0.021]	21.35%
Resilience → Anxiety → Depression → MPA	-0.095	0.023	[-0.141, -0.049]	26.69%

All estimates are standardized coefficients (β). Bootstrap standard errors and 95% bias-corrected confidence intervals were based on 5000 resample.

Although the indirect path via anxiety alone was not statistically significant, the indirect effects via depression and the chained path via anxiety and depression were both significant. These findings support a complete mediation model, suggesting that the mediating factor plays a central role in the relationship between resilience and MPA.

### 3.5 Sensitive analysis

The WLSMV model (**Supplementary Table S2**) showed acceptable fit (CFI = 0.942, TLI = 0.939, RMSEA = 0.052). Path coefficients were consistent in direction and significance with the original ML bootstrap results. Specifically, the indirect effects of resilience on MPA through depression, and the chain pathway remained significant. Effect sizes were comparable, with only minor differences in magnitude (Δβ <.05). This indicates that the conclusions about the mediation effects are robust to different methods of handling ordinal data.

## 4 Discussion

### 4.1 Summary of key findings

**Figure 2:**
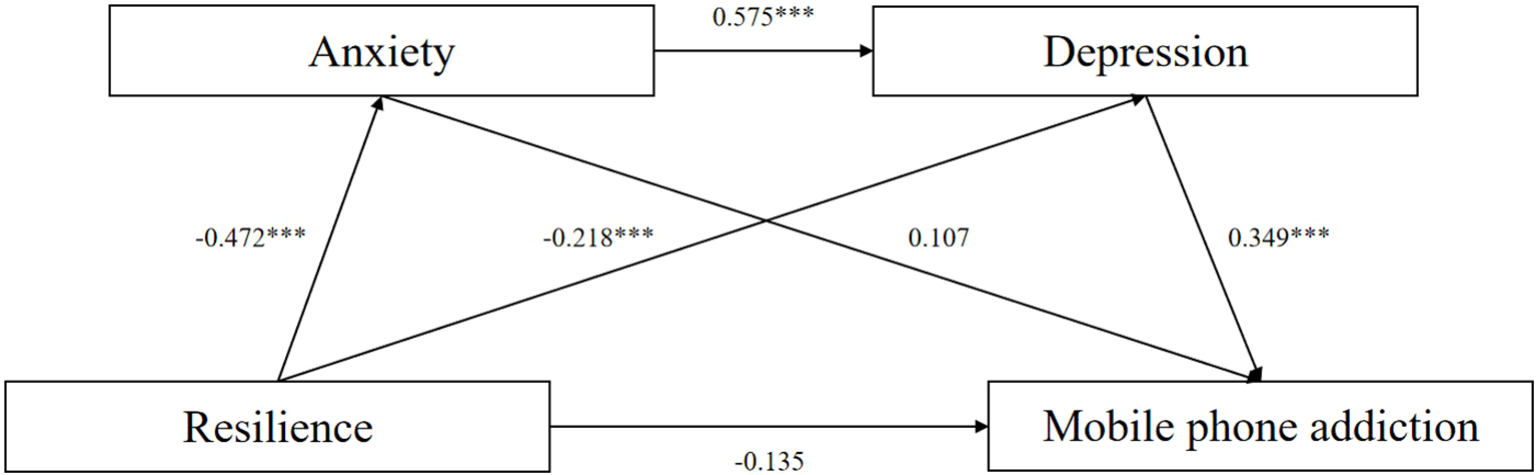
The chain mediation model of Resilience on MPA. *p < 0.05, **p <0.01, ***p < 0.001.

By investigated the relationship between resilience and MPA, focusing on the mediating roles of anxiety and depression. The results partially supported the proposed chain mediation model. Specifically, resilience was negatively associated with both anxiety and depression, while anxiety and depression were positively associated with MPA. Notably, the indirect effects of resilience on MPA through depression alone and through the sequential pathway via anxiety and depression were significant, while the direct path and the anxiety-only mediation were not (**Figure 2**).

### 4.2 Interpretation of individual hypotheses

H1 was not supported, the direct path from resilience to MPA was not significant. This suggests that resilience does not have a direct effect on MPA among adolescent athletes. Previous studies have shown similar results. For example, a study among 257 college students demonstrated that physical activity did not directly reduce MPA but exerted its effect indirectly through enhanced psychological resilience and reduced perceived stress (β = -0.03, p > 0.05) (74). A longitudinal study with 964 Chinese university students found that psychological resilience did not directly predict mobile phone addiction (β = -0.159, p < 0.001) (25). This conclusion is further examined within the population of adolescent athletes. This suggests that the association between resilience and MPA may be more complex and primarily mediated by affective factors rather than direct behavioral regulation, or fully mediated by other factors.

Resilience was significantly and negatively associated with both anxiety (β = -0.472, p < 0.01) and depression (β = -0.218, p < 0.001), indicating that individuals with higher levels of resilience tend to experience lower levels of internalizing symptoms. These findings align with a meta-analysis of 19 studies including 224 middle and late adolescents, which found resilience to be moderately negatively correlated with negative mental health indicators (75). Similarly, anxiety did not significantly predict MPA (β = 0.107, p > 0.05). The active lifestyle and team-based social support of adolescent athletes may reduce anxiety’s direct effect on MPA. Compared to general adolescents, athletes potentially tend to engage in more structured physical activities, experience regular coaching support, and develop higher levels of self-discipline and stress regulation (76).

Depression as a robust positive predictor of MPA (β = 0.349, p < 0.001). For example, a cross-sectional study of 500 Chinese college students found depression significantly positively associated with MPA (r = 0.313, p < 0.001) and a strong predictor of Anxiety (β = 0.377, p < 0.001) (77). H3 was supported as depression mediated the relationship between resilience and MPA, consistent with previous findings (78). H4 was supported, showing anxiety’s strong positive association with depression (79).

### 4.3 Interpretation of mediation effects

The mediation analysis revealed nuanced pathways. While H2 (Resilience → Anxiety → MPA) was not supported, this may be due to potential suppression or overlap effects, which could obscure the true nature of the relationship. Resilience on MPA via depression (H3) was supported, indicating that depression plays an important role as a significant mediator between resilience and MPA. More importantly, H4 was supported: a significant chain mediation path from resilience on MPA through anxiety and depression was observed, highlighting a sequential affective process. This suggests that low resilience may contribute to heightened anxiety, which in turn exacerbates depression, ultimately being associated with higher risk of MPA. These findings emphasize that depression is the emotional mechanism that leads to MPA among adolescent athletes. While anxiety shows consistency but not significance, it might be due to some other factors.

### 4.4 Theoretical contributions

This study contributes to the broader understanding of MPA by integrating resilience, anxiety, and depression into a single framework. The findings extend the I-PACE model by positioning resilience as a distal personal factor and identifying anxiety and depression as proximal affective driver of MPA. The significance of the chain mediation further supports the affective pathway model, suggesting that behavioral addictions of teenager athletes may result from cascading emotional disturbances.

### 4.5 Practical implications

The findings provide several practical implications for the prevention of MPA among adolescents, particularly those engaged in regular physical training.The results suggest that resilience is associated with a lower risk of MPA, primarily through its effect on depressive symptoms. Thus, focusing on resilience may be an effective strategy for intervening in MPA behavior. Since depression emerged as the critical mediator, targeted interventions focusing on early identification and treatment of depressive symptoms are important. Identifying effective strategies within schools may help address negative emotion before they develop into addictive behaviors. The results underscores the importance of recognizing the sequential emotional pathway from anxiety to depression to MPA. It suggests that interventions should not treat emotional problems in isolation but adopt a multi-layered approach that addresses co-occurring symptoms holistically. For adolescents in performance-oriented environments, such as sports schools, integrating resilience development into athletic training may be an effective strategy for addressing MPA.

### 4.6 Limitations

Despite its contributions, this study has limitations. The cross-sectional design precludes causal inference. Longitudinal or experimental designs are needed to confirm the causality of the relationships. Furthermore, the sample sizes of this study were all from physical education middle schools. The original resilience of these sample sizes might be stronger than that of ordinary teenagers (80), and their various psychological qualities were all relatively high (81), resulting in the data not being potentially applicable to teenagers who lack physical exercise. It is also possible to expand the sample size to include ordinary teenagers and incorporate a scale for measuring physical exercise would enhance the generalizability of these findings. Moreover, we employed Harman’s single factor test to assess common method bias. However, this approach has limitations, including its assumption of a single underlying factor and its potential to overlook certain sources of bias, which may limit the robustness of the results. And all data were collected through self-report measures without objective assessments, which could introduce potential sources of bias and instability. The path from resilience to MPA through anxiety may reflect suppression or overlap, which could explain the non-significant association found. Three items exhibited relatively substandard factor loadings, suggesting potential measurement issues in this model. Future research may consider re-examining these items or using alternative measures to enhance construct validity. Future research should explore the role of other emotional or cognitive mediators in the resilience and MPA link.

### 4.7 Conclusion

(1) Resilience was significantly associated with MPA through complete mediators among adolescents, although the direct path was not significant. (2) The mediating path from resilience to MPA through depression was significant, while the path through anxiety was not. (3) The chain mediation path from resilience to MPA from anxiety and depression was significant. These findings highlight the indirect role of emotional symptoms in linking resilience to MPA among adolescent athletes, suggesting that enhancing resilience could be an effective strategy for intervening the risk of MPA in this specific population.

## Data Availability

The original contributions presented in the study are included in the article/**Supplementary Material**. Further inquiries can be directed to the corresponding authors.
