# Supplementary material for: Resilience and mobile phone addiction among adolescent athletes: a chain-mediated model of anxiety and depression
Source: Front Psychiatry. 2025 Nov 28;16:1646150. doi: 10.3389/fpsyt.2025.1646150 (PMC12699149; doi:10.3389/fpsyt.2025.1646150)
Supplement: Supplementary file 1 [file Table1.doc]

**Table S1.** CFA factor loading

| **Latent Variable** | **Item** | **Std. Loading** | **SE** | **p-value** |
| --- | --- | --- | --- | --- |
| **Anxiety** | C1 | 0.664 | 0.022 | < .001 |
| **Anxiety** | C2 | 0.681 | 0.026 | < .001 |
| **Anxiety** | C3 | 0.704 | 0.027 | < .001 |
| **Anxiety** | C4 | 0.779 | 0.026 | < .001 |
| **Anxiety** | C5 | 0.681 | 0.028 | < .001 |
| **Anxiety** | C6 | 0.705 | 0.027 | < .001 |
| **Anxiety** | C7 | 0.656 | 0.03 | < .001 |
| **Depression** | D1 | 0.654 | 0.031 | < .001 |
| **Depression** | D10 | 0.767 | 0.028 | < .001 |
| **Depression** | D11 | 0.496 | 0.044 | < .001 |
| **Depression** | D12 | 0.441 | 0.045 | < .001 |
| **Depression** | D13 | 0.545 | 0.039 | < .001 |
| **Depression** | D14 | 0.636 | 0.035 | < .001 |
| **Depression** | D15 | 0.571 | 0.029 | < .001 |
| **Depression** | D16 | 0.421 | 0.045 | < .001 |
| **Depression** | D17 | 0.438 | 0.025 | < .001 |
| **Depression** | D18 | 0.706 | 0.033 | < .001 |
| **Depression** | D19 | 0.737 | 0.027 | < .001 |
| **Depression** | D2 | 0.468 | 0.034 | < .001 |
| **Depression** | D20 | 0.282 | 0.039 | < .001 |
| **Depression** | D3 | 0.608 | 0.033 | < .001 |
| **Depression** | D4 | 0.257 | 0.053 | < .001 |
| **Depression** | D5 | 0.65 | 0.037 | < .001 |
| **Depression** | D6 | 0.783 | 0.032 | < .001 |
| **Depression** | D7 | 0.763 | 0.031 | < .001 |
| **Depression** | D8 | 0.334 | 0.052 | < .001 |
| **Depression** | D9 | 0.732 | 0.032 | < .001 |
| **MPA** | E1 | 0.458 | 0.054 | < .001 |
| **MPA** | E10 | 0.682 | 0.05 | < .001 |
| **MPA** | E11 | 0.651 | 0.054 | < .001 |
| **MPA** | E12 | 0.739 | 0.048 | < .001 |
| **MPA** | E13 | 0.636 | 0.047 | < .001 |
| **MPA** | E14 | 0.66 | 0.05 | < .001 |
| **MPA** | E15 | 0.656 | 0.047 | < .001 |
| **MPA** | E16 | 0.741 | 0.049 | < .001 |
| **MPA** | E2 | 0.651 | 0.051 | < .001 |
| **MPA** | E3 | 0.585 | 0.05 | < .001 |
| **MPA** | E4 | 0.758 | 0.045 | < .001 |
| **MPA** | E5 | 0.715 | 0.049 | < .001 |
| **MPA** | E6 | 0.76 | 0.048 | < .001 |
| **MPA** | E7 | 0.742 | 0.046 | < .001 |
| **MPA** | E8 | 0.739 | 0.052 | < .001 |
| **MPA** | E9 | 0.663 | 0.049 | < .001 |
| **Resilience** | F1 | 0.695 | 0.043 | < .001 |
| **Resilience** | F10 | 0.695 | 0.044 | < .001 |
| **Resilience** | F2 | 0.816 | 0.038 | < .001 |
| **Resilience** | F3 | 0.75 | 0.044 | < .001 |
| **Resilience** | F4 | 0.775 | 0.04 | < .001 |
| **Resilience** | F5 | 0.763 | 0.041 | < .001 |
| **Resilience** | F6 | 0.826 | 0.038 | < .001 |
| **Resilience** | F7 | 0.776 | 0.04 | < .001 |
| **Resilience** | F8 | 0.712 | 0.046 | < .001 |
| **Resilience** | F9 | 0.819 | 0.041 | < .001 |

Notes.
All factor loadings are standardized estimates. SE = Standard Error. All p-values < .001. Items are retained without parceling to preserve scale structure.

**Table S2.** WLSMV results of chain mediating effect of Resilience on MPA

| **Effect** | **Effect Size (β)** | **SE** | **95% CI [LL, UL]** | **Proportion mediated** |
| --- | --- | --- | --- | --- |
| **Total effect** | -0.366 | 0.042 | [-0.449, -0.283] | — |
| **Direct effect** | -0.082 | 0.058 | [-0.196, 0.032] | — |
| **Total indirect effect** | -0.284 | 0.033 | [-0.349, -0.220] | 77.60% |
| **Resilience → Anxiety → MPA** | -0.045 | 0.038 | [-0.119, 0.029] | 12.30% |
| **Resilience → Depression → MPA** | -0.131 | 0.029 | [-0.187, -0.075] | 35.79% |
| **Resilience → Anxiety → Depression → MPA** | -0.108 | 0.021 | [-0.149, -0.066] | 29.50% |

*Notes*.
All estimates are standardized coefficients (β).
